# Misalignment in South African midwife specialists’ education and practice legal frameworks

**DOI:** 10.4102/curationis.v48i1.2740

**Published:** 2025-12-10

**Authors:** Kagiso P. Tukisi

**Affiliations:** 1 Department of Nursing, Faculty of Health Sciences, University of Johannesburg, Johannesburg, South Africa

**Keywords:** education and training, independent function, legislative framework, midwife

## Abstract

**Background:**

The midwifery specialisation is one of sixteen clinical nursing specialisations in South Africa aimed at ensuring safe and comprehensive patient care. While specific training guidelines to ensure expanded knowledge and skills amongst the midwife specialists exist, there are no relevant regulations to legalise their specialists’ knowledge. Current practice regulations are unsupportive of the midwife specialists’ roles in clinical settings.

**Objectives:**

This study aimed to explore and describe the legislative framework of midwife specialists’ education and practice through the lens of midwife specialists in South Africa.

**Method:**

Qualitative, explorative and descriptive research was followed. Sixteen midwife specialists identified through purposive sampling participated in virtual, semi-structured focus group interviews. Data were analysed using Collaizi’s descriptive method. The context of the study is the public maternal health services of South Africa.

**Results:**

One central theme with seven subthemes emerged. Midwife specialists verbalised their views on the current education and training regulations (R.212; R.635 and competencies of midwife specialists). The midwife specialists also shared their views on practice regulations (R.2127, R.2488, R.767 and position statements on registered midwives’ allocation and participation in surgical procedures). Additionally, midwife specialists highlighted the misalignments in these regulations.

**Conclusion:**

Revision of the current legislation must align the midwife specialists’ education and training competencies and outcomes. The alignment of these regulations will in turn legalise their specialists’ practice.

**Contribution:**

This article advocates for the regulatory body’s legal recognition of midwife specialists to enable autonomous and independent practice among midwife specialists.

## Introduction

Midwife specialist training has existed for over four decades in South Africa (Sellers et al. 2018). The midwife specialist training was a response measure to the escalating maternal and neonatal mortalities in an era where a shortage of doctors was prevalent (Sellers et al. 2018). The obstetricians empowered the midwives with what was previously referred to as advanced obstetric skills in 1980 to strengthen the multidisciplinary teams (Mativandlela 1998). The South African Nursing Council (SANC) formulated, approved and promulgated the generic education and training regulations for midwives, leading to registration as a midwife specialist (R212) in 1993 (SANC 1993). R212 was repealed and replaced by education and training regulations for midwives, which led to their registration as midwife specialists (R635) in 2020 (SANC 2020).

The education and training regulations were derived from the *Nursing Act number 50* stipulations of 1978, which was amended by the *Nursing Act 33 of 2005* (SANC 2005). Chapter 2, Sections 30 and 32 of the *Nursing Act 33 of 2005* grant SANC the powers as a professional regulatory body to safeguard the education, training and practice of the nursing and midwifery profession in South Africa (SANC 2005). A midwife specialist is a registered nurse with advanced midwifery expertise who is registered with the SANC (SANC 2014). A midwife specialist is a senior midwifery professional with extensive knowledge, experience and skills who has undergone training that extends beyond that of a registered midwife (Toll et al. 2024). The midwifery training in South Africa was guided by training regulations 425 and R254, which led to registration as a midwife before 2020 (SANC 1985, 1993). After 2020, midwifery training will be attained under the prescripts of the new regulations 174 and 1497 (Department of Health [DOH] 2019; SANC 2014).

The midwives’ training provides the registered midwives with sufficient knowledge and skills to care for and support women, newborns and families throughout the antenatal, intrapartum, postnatal and neonatal periods (Kennedy et al. 2020). The registered midwife provides midwifery care services in various clinical facilities, including primary healthcare clinics, midwife-led obstetric units (MOU) and hospitals (Kennedy et al. 2020). Registered midwives provide knowledge to women, families and communities through counselling on antenatal education, preparation for parenthood and sexual and reproductive healthcare (DoH 2019). Additionally, registered midwives are responsible for assessing, classifying, managing and referring high-risk patients to physicians and hospitals (Tavakol et al. 2025). Postgraduate midwifery studies aim to expand registered midwives’ knowledge and skill sets to enable midwife specialists to manage complicated obstetric and neonatal conditions too complex for registered midwives.

A prospective applicant must hold an undergraduate nursing qualification to qualify for admission as a postgraduate midwifery student in South Africa. Additionally, a registered midwife requires at least 2 years of clinical experience in the midwifery field (Crowley & Daniels 2023). Additionally, a prospective midwife must register with the SANC as a registered midwife (Crowley & Daniels 2023). During training, a postgraduate midwifery student must complete a theoretical component of the postgraduate midwifery studies and a minimum of 1000 clinical hours in clinical practice for experiential learning (DoH 2019; SANC 2014).

Postgraduate midwifery studies embed knowledge and skills such as advanced expertise in midwifery and neonatal nursing care, guided by heightened clinical judgement (SANC 2014). Consequently, midwife specialists can detect maternal and neonatal complications and carry out emergency clinical interventions prior to referrals to higher levels of care (Gettings 2024). The midwife specialists’ expertise in midwifery positions a midwife specialist as a leader and an advocate in the reproductive, maternal and neonatal healthcare context. Furthermore, the expanded knowledge and skills position a midwife specialist as a collaborator and a health team member to benefit maternal and neonatal nursing clients (Gettings 2024). The expansion of midwife specialists’ knowledge and skills aligns with the World Health Organization’s (WHO) recommendation of task-shifting between midwives and physicians to increase access to and efficiency of maternal and neonatal health services (WHO 2022). A similar phenomenon has been reported in countries such as Uganda and Liberia, where midwives’ knowledge expands without the relevant legislative framework to guide the application of such knowledge and skills in practice (Nygaard Moller 2024).

### Problem statement

The midwife specialist profession has existed for three decades since its approval by the SANC. However, midwife specialists’ clinical roles and responsibilities must be better understood in the clinical practice environment. The misunderstanding of the midwife specialists’ roles and responsibilities in clinical practice dates to 1998, 5 years after SANC approved the programme in 1993 (Mativandlela 1998). The existing literature highlights the need for more specific regulations from the SANC detailing the specific roles and responsibilities of the midwife specialists as they are empowered through formal and extracurricular trainings, such as essential steps in the management of obstetrical emergencies and obstetric ultrasound (Lukhele et al. 2023; Tukisi et al. 2025). To date, midwife specialists are unable to transition from the registered midwives to the midwife specialists’ role. Consequently, both registered midwives and midwife specialists seem to perform similar roles and responsibilities, resulting in role confusion. The absence of specific practice regulations, such as the scope of practice and practice guidelines, hindered autonomous and independent practice (Lukhele et al. 2023). The researcher is a midwife specialist with clinical and midwifery education experience and identified gaps in the education and practice of midwife specialists.

## Research methods and design

A qualitative, exploratory and descriptive research design was followed to explore and describe the midwife specialists’ education, training and legislative framework from the midwife specialists’ perspectives. The explorative and descriptive research design was relevant to uncovering information on the phenomenon under study.

### Setting

The study took place in the public sector clinical facilities within the seven provinces of South Africa. The public sector is pivotal in providing essential maternal and neonatal healthcare services to 70% of South African citizens. The midwife specialists’ practice is guided by the education and practice regulations from SANC and the guidelines provided by the Department of Health.

### Study population

The population comprised midwife specialists employed in the public health sector of South Africa. Participants were required to hold additional qualifications in midwifery and registration with SANC as midwife specialists. Additionally, participants were required to have at least 3 years of experience as midwife specialists and be willing to participate in the study. Using purposive sampling, a researcher recruited midwife specialists via the nursing services managers of various facilities, who were gatekeepers. The gatekeepers introduced the researcher to the participants and shared the research information letters. Interested participants made telephone contact with the researcher and shared their email addresses. The researcher made it a point to keep the participants’ contact details confidential and restricted communication of research information in line with the *POPIA Act (Protection of Personal Information Act 4 of 2013)*. The researcher selected a sample of midwife specialists using the following inclusion criteria.

### Inclusion criteria

The study included participants who met the following criteria:

- Registered with the South African Nursing Council (SANC) as a midwife specialist.
- Currently employed in public healthcare facilities (clinics, maternal obstetric units or hospitals).
- Possessed a minimum of 3 years of professional experience working specifically as a midwife specialist.

### Exclusion criteria

Individuals were excluded from participation if they:

- Had less than 2 years of experience practising as a midwife specialist.
- Were not currently employed in public healthcare facilities (clinics, MOUs, or hospitals).
- Were not registered with SANC as midwife specialists.

The researcher aimed to conduct five focus groups comprising four participants each. However, data saturation was reached during the third focus group interview. The researcher conducted a fourth focus group interview to confirm the saturation. The study comprised 16 midwife specialists (P1–P16) who participated in four focus group discussions, each containing four participants. All participants were highly qualified healthcare professionals with specialised midwifery and neonatal care training. Each participant held the post-basic midwifery and neonatal nursing qualification (R212), demonstrating their advanced maternal and newborn healthcare expertise.

The participants’ educational backgrounds revealed a mix of diploma and degree qualifications. More than two-thirds of the participants (68.8%) entered the profession with a Diploma in nursing and midwifery (R425), while nearly a third (31.2%) held bachelor’s degrees in nursing and midwifery (R425). Two participants followed an alternative educational pathway, obtaining a Diploma in Nursing (R683) before completing a separate Diploma in midwifery (R254).

Many participants had pursued further academic advancement beyond their basic professional qualifications. Six participants (37.5%) had enhanced their clinical expertise with additional bachelor’s degrees in nursing education and administration. In comparison, two participants (12.5%) had achieved master’s level qualifications in post-basic midwifery and neonatal nursing. This diversity in educational preparation enriched the focus group discussions with perspectives informed by various levels of academic training and specialisation in maternal and newborn care.

These participants demonstrated considerable professional experience in midwifery, with years of service ranging from 6 to 18 years and an average of 10.2 years in the field. Most participants (62.5%) had practised midwifery for 8–12 years, providing a solid foundation of clinical expertise, while two senior practitioners contributed more than 15 years of experience to the discussions.

Participants worked across various healthcare settings, offering perspectives from different levels of maternal care services. Half of the participants (eight midwives) were based in hospitals, while more than a third (six midwives) worked in midwife obstetric units (MOUs). The remaining two participants practised in clinic settings, ensuring representation of the full spectrum of maternal healthcare facilities where obstetric ultrasound referrals commonly occur.

The study achieved substantial geographic diversity, drawing participants from seven of South Africa’s nine provinces. The Northern Cape and Limpopo provinces had the highest representation, with three participants each, accounting for 37.6% of the total sample. The remaining provinces – Gauteng, North West, Eastern Cape, Free State, and KwaZulu-Natal – were equally represented with two participants. The demographic data of the participants are summarised in Table 1.

**TABLE 1:** The participants’ demographic data.

Participant number	Qualifications	Years of experience	Public facility	Province
**Focus group interview group 1**				
P1	Bachelor of nursing and midwifery (R425) Master’s in post-basic midwifery and neonatal nursing (R212)	08	MOU	North West
P2	Diploma in nursing and midwifery (R425) Bachelor of nursing education and administration Master’s in post-basic midwifery and neonatal nursing (R212)	10	Hospital	Eastern Cape
P3	Diploma in nursing and midwifery (R425) Diploma in post-basic midwifery and neonatal nursing (R212)	11	MOU	Gauteng
P4	Bachelor of Nursing and Midwifery (R425) Diploma in post-basic midwifery and neonatal nursing (R212)	07	Hospital	Limpopo
**Focus group interview group 2**				
P5	Bachelor of nursing and midwifery (R425) Master’s in post-basic midwifery and neonatal nursing (R212)	09	Hospital	Limpopo
P6	Diploma in nursing and midwifery (R425) Diploma in post-basic midwifery and neonatal nursing (R212)	11	Hospital	Free State
P7	Diploma in nursing and midwifery (R425) Diploma in post-basic midwifery and neonatal nursing (R212)	08	Hospital	KwaZulu-Natal
P8	Diploma in nursing and midwifery (R425) Bachelor of nursing education and administration with post-basic midwifery and neonatal nursing (R212)	06	MOU	Northern Cape
**Focus group interview group 3**				
P9	Diploma in nursing and midwifery (R425) Diploma in post-basic midwifery and neonatal nursing (R212)	09	Clinic	Gauteng
P10	Diploma in nursing and midwifery (R425) Bachelor of nursing education and administration with post-basic midwifery and neonatal nursing (R212)	07	MOU	Limpopo
P11	Diploma in nursing (R.683) Diploma in midwifery (R254) Diploma in post-basic midwifery and neonatal nursing (R212)	16	Clinic	North West
P12	Bachelor of Nursing & Midwifery (R425) Diploma in post-basic midwifery and neonatal nursing (R212)	13	MOU	Northern Cape
**Focus group interview group 4**				
P13	Diploma in nursing & midwifery (R425) Bachelor of nursing education and administration with post-basic midwifery and neonatal nursing (R.212)	09	Hospital	Free State
P14	Bachelor of Nursing and Midwifery (R425) Diploma in post-basic midwifery and neonatal nursing (R212)	11	Hospital	Northern Cape
P15	Diploma in nursing and midwifery (R425) Bachelor of nursing education and administration with post-basic midwifery and neonatal nursing (R212)	12	MOU	Eastern Cape
P16	Diploma in nursing (R683) Diploma in midwifery (R.254) Diploma in post-basic midwifery and neonatal nursing (R.212)	18	Hospital	KwaZulu-Natal

*Source*: Adapted from Tukisi, K.P., Janse Van Rensburg, Z. & Jacobs, W., 2024, ‘South African midwife specialists’ experiences in the utilisation of their knowledge and skills’, *Health SA Gesondheid* 29, 2444. https://doi.org/10.4102/hsag.v29i0.2444

MOU, midwife-led obstetric units; P, participant.

### Data collection

Data collection took place from February 2022 to July 2022. Before the data collection, approval from the University of Johannesburg (UJREC), the provincial health department and respective facilities was sought. The chief executive officers, including nursing services managers, provided permission to access the midwife specialists. The contact details that the prospective participants shared during the recruitment process were used to circulate the links for Microsoft Teams scheduled at a date and time convenient for the participants. Despite the virtual nature of the Microsoft Teams platform and participants’ cameras being turned off, careful attention was paid to vocal modulations, speech patterns, emphasis and tonal variations. These paralinguistic elements provided valuable contextual data that complemented the verbal content of participant responses. Research information letters, consent forms for participation and recordings of the interviews were shared to gain informed consent.

The participants formed groups of midwife specialists from different provinces for the semi-structured focus group interviews (FGI). The midwife specialists initiated the FGI, sharing their demographic data pertinent to their inclusion in the study. The interview guide comprised a central question:

*‘What is your perspective on the legislative framework of midwife specialists’ education and practice in South Africa?’*

Probing questions were used to direct the flow of conversations and gain in-depth information from the participants regarding the phenomenon under study (Gerson & Damaske 2020). The interview lasted between 60 min and 90 min, and participants had an equal chance to share their experiences through the moderator. The first FGI was regarded as the pilot study to assess the need to refine the research questions. However, the research question was understandable, as participants were able to respond to the question without challenges. Consequently, the first FGI was included in the main sample. Data saturation was reached during the third FGI, as evidenced by the lack of new data and redundancy of information from the participants. The researcher conducted the fourth FGI to confirm the saturation.

### Data analysis

Data from the FGI were transcribed using the Microsoft Teams automated voice transcriptions. However, the researcher made it a point to validate the transcriptions by listening to the audio against the transcriptions to ensure accuracy. Data were analysed using Collaizi’s steps of qualitative data analysis outlined in Polit and Beck (2024). The researcher intensely read the transcriptions, self-immersed in data and extracted the significant statements. The significant statements were clustered into themes and categories detailing the midwife specialists’ perspectives on the education, training and practice legislative framework. The service of an independent coder was sought, and a consensus meeting was held to agree on the themes and categories. The analysed data were conceptualised and integrated into the existing literature.

### Ethical considerations

The researcher was cognisant that the study involved human participants. Therefore, a proposal for health science research adherent to health science research ethics was developed and submitted to the University of Johannesburg. The ethical clearance was received from the University of Johannesburg, Faculty of Health Sciences Research Ethics Committee and Higher Degrees Committee (reference numbers: REC-1279-2021). Regarding participants’ right to justice, the research information letters were circulated in writing to gain the participants’ voluntary and informed consent. An inclusion criterion detailed in the approved research proposal was used to select the participants, which ensured a just and fair selection (Moriña 2021). Privacy and confidentiality were ensured by generating codes specific for the discussion of data, which prevented participants from being linked to the data, thus ensuring anonymity. Additionally, data were kept in a password-encrypted file accessible to the researcher, which maintained confidentiality.

### Trustworthiness

The study’s rigour was ensured and established using the five principles of Lincoln and Guba (1978), cited in Polit and Beck (2024). The researcher kept a transparent record of the research processes in the form of an audit trail, thus strengthening the accuracy and reliability of the data, which increased the study’s credibility (Adler, 2022). Additionally, the researcher performed a preliminary data analysis independently and employed an independent coder. Themes and categories were discussed, which ensured data analysis from multiple perspectives, thus increasing the quality of data (Polit & Beck, 2024). To ensure the study’s dependability, the researcher used the code-recode method. To ensure dependability, code-recode and co-coding methods were used to compare analyses from the exact transcriptions, thus ensuring consistency (Adler, 2022). The researcher described the research methods and participants’ demographic information pertinent to their selection and participation in the study, thus increasing the transferability and generalisability of the study (Polit & Beck, 2024). The researcher documented all the relevant data analysis information, including audio recordings, written notes and transcriptions, ensuring the study’s confirmability (Hayre 2021). To ensure authenticity, the researcher selected the participants using the inclusion and exclusion criteria approved for the study. The participants were selected using the study’s approved inclusion and exclusion criteria, which ensured fairness and excluded selection bias. Additionally, the participants’ emotions were captured, which enriched the data and thus increased the study’s authenticity (Hayre 2021).

## Results

One central theme detailed in Table 2 emerged as follows: midwife specialists highlighted that there are specific education and training regulations from SANC detailing the level of competencies to be achieved. However, the current regulations guiding the midwife specialists from SANC need to be aligned with the midwife specialists’ knowledge, skills and competencies. Consequently, the midwife specialists’ practice needs to be aligned with their education and training level. Midwife specialists recommend revising the current legislative framework, thus ensuring alignment between education, training and professional practice.

**TABLE 2:** Summary of description of themes and categories.

Overarching theme	Categories
1. Misalignment between midwife specialists’ education, training and practice regulation	R.635 of 05 June 2020 Regulations relating to the approval and the minimum requirements for the education and training of a student leading to registration as a Nurse Specialist or a Midwife Specialist Competencies of Midwife Specialists R.2127 of 03 June 2022 Regulations regarding the Scope of Practice for Nurses and Midwives R.2488 of 26 October 1990 Regulations relating to the conditions under which Registered Midwives and Enrolled Midwives may carry out their profession R.767 of 01 October 2014 Regulations setting out the acts or omissions in respect of which the Council may take disciplinary steps SANC – Position paper on the use of Registered Nurses and Midwives as assistant surgeons SANC – Position paper on the allocation of non-specialised nurses in specialised units

SANC, South African Nursing Council.

### Theme 1: Misalignment between midwife specialists’ education, training and practice regulation

#### Category 1: R.635 of 5 June 2020 Regulations relating to the approval and the minimum requirements for the education and training of a student leading to registration as a nurse specialist or a midwife specialist

The midwife specialists confirmed their awareness of an approved curriculum that guides the training of midwife specialists. They highlighted that the training received through midwife specialists’ education and training curriculum surpasses that of registered midwives. The midwife specialist elaborated:

‘[*I*]f you are going to look at the curriculum and education being provided to an advanced midwife, it allows you to be beyond and be above a basic midwife.’ (P11, 16 years, clinic, North West)

The midwife specialists posited that they gained clinical knowledge and skills to attend to severe obstetrical complications during their specialists’ training:

‘I am a specialist by certificate, but when it comes to my function, apart from delivering a breech, I am also skilled in assisted deliveries, forceps, and vacuums.’ (P9, 09 years, Clinic, Gauteng)

#### Category 2: Competencies for midwife specialists

The midwife specialists highlighted that, in addition to the prescribed and approved training regulations for midwives, there are competencies that describe knowledge and skills that can be embedded in a prospective midwife specialist during training. The midwife specialist mentioned:

‘I rely mainly on the competencies of an advanced midwife that confirm that I have advanced knowledge and skills above advanced midwifery.’ (P3, 11 years, MOU, Gauteng)

According to the midwife specialists, SANC, as a regulatory body, is aware of the contents of the midwife specialists’ training and the related competencies. Therefore, SANC defines a midwife specialist in terms of the competencies and the characteristics. Midwife specialists elaborated:

‘SANC also approved a curriculum for us in the universities and colleges, so they know exactly what we are studying as advanced midwives and the competencies they expect from us.’ (P7, 08 years, Hospital, KwaZulu-Natal)

#### Category 3: R.2127 of 03 June 2022 regulations regarding the scope of practice for nurses and midwives

The midwife specialists knew that upon completing their postgraduate midwifery studies, which led to their registration as midwife specialists, they needed to be guided by the scope of practice. However, midwife specialists highlighted that there is yet to be an existing scope of practice that specifies the roles and responsibilities of midwife specialists. The midwife specialists elaborated:

‘At the end of practice, when one has graduated, you are supposed to adhere to a particular scope of practice, but currently, there is none for us advanced midwives.’ (P1, 08 years, MOU, North West)

The midwives reiterated that they needed to be aware of the scope of practice so that they could practise as outlined by the scope of practice. Midwife specialists explained:

‘I am an advanced midwife. I need to know that I am well within my rights and scope of practice to do that.’ (P4, 12 years, MOU, Eastern Cape)

The midwife specialists highlighted that under the current scope of practice, they can only openly practise some of the knowledge and skills embedded during the specialist’s training. The midwife specialists believed that the absence of the specific roles and responsibilities of midwife specialists within the current scope of practice suggests that they are not legally permitted to apply such knowledge and skills. The midwife specialists elaborated:

‘By practicing on the sly, I mean that if all those interventions I know of because I have studied them during my training do not appear on the scope of practice and hospital policies to authorise my practice, then I am acting outside of what I am authorised to do.’ (P2, 10 years, Hospital, Eastern Cape)

#### Category 4: R.2488 of 26 October 1990 regulations relating to the conditions under which registered midwives and enrolled midwives may carry out their profession

The midwife specialists knew that the conditions under which the midwives carry out their profession are an extension of the scope of practice. However, they were troubled by the fact that the regulations have no specific reference to the conditions under which the midwife specialists carry out their profession. The midwife specialist explained:

‘The one we are using is that of essential midwives R.2488; there is no mention of advanced Midwives in that one.’ (P16, 18 years, Hospital, KwaZulu-Natal)

The midwife specialists reiterated that the exclusion of the specifications to guide the midwife specialists subjected them to assuming their previous midwifery roles and responsibilities. The midwife specialist mentioned:

‘I returned to the same job description; my rank was the only thing I saw change. I was translated from professional nurse to professional nurse specialty nursing. So I got back to do the very same job.’ (P12, 13 years, Hospital, Northern Cape)

#### Category 5: R.767 of 01 October 2014 regulations setting out the acts or omissions in respect of which the council may take disciplinary steps

The midwife specialists knew the potential risk of litigation and sanctions that the SANC may institute. The midwife specialists expressed that their specialist knowledge and skill set, as well as the absence of a specific scope of practice, make it challenging to remain within the legal boundaries. Consequently, concerns have been raised about unauthorised actions on the part of the midwife specialists. The midwife specialist elaborated:

‘Sometimes, something can go wrong with procedures, and we have undesired outcomes! So obviously, they are going to pull out the scope of practice, and you are in for it and asked if you have overstepped the mark, so how do you account for that? In a nutshell, nothing protects me as an advanced midwife.’ (P8, 06 years, MOU, Northern Cape)

The midwife specialists highlighted that sometimes, remaining within the professional boundaries of the scope of practice raises concerns about omissions. The midwife specialists highlighted that in case of serious complications, they run a risk of sanctions by omitting interventions that may have contributed positively to the patient’s outcomes. The midwife specialist elucidated:

‘If you also try to work under the scope of practice and avoid the advanced interventions, then everyone is too quick to remind you of your advanced knowledge and skill. They will remind you of that and ask again, “Why didn’t you assist the patient?”.’ (P6, 10 years, Hospital, Free State)

The midwife specialists found themselves in a quandary during emergencies at the risk of accountability for either a risk or an omission. The midwife specialist mentioned:

‘So eish, it is a serious dilemma; we are not supposed to go through this.’ (P13, 09 years, Clinic, Gauteng)

#### Category 6: SANC – Position paper on the use of registered nurses and midwives as assistant surgeons

The midwife specialists highlighted that some of the skills embedded during the midwife specialists’ training were invasive and included assisting physicians intraoperatively. The midwife specialists mentioned that they could not utilise such knowledge and skills. The midwife specialists elaborated:

‘We are also trained to scrub in theatre and assist with the surgery itself, but that is limited strictly to the workbook; you will never do it again after completing the course in clinical practice.’ (P14, 11 years, Hospital, Northern Cape)

‘We are also able to act as assistant surgeons! We have been equipped with that knowledge, but it is only used in some places.’ (P10, 07 years, MOU, Limpopo)

According to the midwife specialists, their inability to utilise their knowledge and skills stems from direct communiques from SANC that caution against professional acts outside of the scope of practice. Midwife specialists mentioned:

‘SANC forbade the participation of nurses and midwives in assisting surgeons, even for minor surgeries. Such procedures are invasive. All invasive procedures are not covered by the scope of practice.’ (P8, 06 years, MOU, Northern Cape)

#### Category 7: South African Nursing Council – Position paper on the allocation of non-specialised nurses in specialised units

The midwife specialists highlighted that the SANC advocates for nurses and midwife specialists to be allocated in their areas of specialisation. The midwife specialists were aware of this necessity as the emergence of serious complications required specialists’ advanced knowledge and skills, especially in the absence of physicians. The midwife specialist elucidated:

‘All four of our teams have an advanced midwife who is a team leader! They usually coordinate resuscitations and interventions in emergencies because the doctors are sometimes held up when we need them. However, we need to help the patient at that time.’ (P3, 11 years, MOU, Gauteng)

Although the midwife specialists were satisfied with the recommendation from the SANC, they were also concerned that no legislative framework justified their allocation in the specialised midwifery units. The midwife specialists highlighted a need for regulations detailing their roles and responsibilities in managing maternal and neonatal complications. Midwife specialists elucidated:

‘We also have experienced midwives doing a great job managing all these complications! Nevertheless, the problem is that there are just no policies telling us this is what we must do, but then we know that we have to try and save the lives of mothers and their babies.’ (P15, 12 years, MOU, Eastern Cape)

#### Category 8: Recommendations for review of all the regulations to include midwife specialists

With the specialist’s knowledge and skills according to the prescribed education and training regulations, midwife specialists recommended that the current legislative framework guiding their practice be revised. The midwife specialists reiterated that the scope of practice requires revision because it is a legal document that provides legal parameters for their professional practice. Midwife specialists elaborated:

‘The job description is informed by the scope of practice, which should be from the South African Nursing Council! According to us, SANC should say that the midwife who has been prepared under that specific regulation is supposed to do this and this and that. The advanced midwife is capable of doing 1, 2, and 3. Therefore, these are the parameters the advanced midwife should practice under or within.’ (P9, 09 years, Clinic, Gauteng)

The midwife specialists reiterated that revising the scope of practice would provide reassurance of legal protection during the management of severe complications, and they can aid their juniors. The midwife specialists explained:

‘So, policies must be reviewed to practice knowing that I am legally covered to do this. Even the junior midwives will know that they can come to me for help if they have diagnosed a serious complication.’ (P7, 08 years, Hospital, KwaZulu-Natal)

The midwife specialists echoed that revising the scope of practice will draw a clear distinction between midwife specialists and registered midwives and increase their independent and autonomous practice. Midwife specialists elucidated:

‘If we do have our scope of practice, we will be able to practice independently; it would not matter if the doctor is here; I will continue to perform my duties without worrying about overstepping the mark and calling the doctor to help.’ (P5, 09 years, Hospital, Limpopo)

‘These protocols should include more than just an ordinary midwife; they must include the advanced midwife. If policies could be inclusive and more specific on the roles and responsibilities of the midwives and advanced midwives, that would help.’ (P3, 11 years, MOU, Gauteng)

## Discussion

The study sought to explore and describe the legislative framework of midwife specialists’ education and practice through the lens of midwife specialists in South Africa. The midwife specialists confirmed that there is an existing and approved curriculum for the education and training of midwife specialists in South Africa. According to the literature, midwife specialists in South Africa were trained under Regulation 212 prior to phasing out of the qualification post-basic midwifery and neonatal nursing in 2016 (SANC CIRCULAR;7/2012). The post-basic midwifery and neonatal nursing were replaced by the postgraduate diploma in midwifery under regulation 635 in 2020 (SANC 2020). Notably, these education and training regulations emphasise the expansion of knowledge and skills of essential midwives in line with the competencies of midwife specialists (SANC 2013). The regulation of education and training is a prerogative of the SANC as determined by the *Nursing Act 33 of 2005*. Upon completing the postgraduate midwifery training, the midwife specialists’ qualification is registered as an additional qualification with the SANC under section 34 of the Act (SANC 2005).

Although there is an approved curriculum and set of competencies for midwife specialists, concerns remain regarding the current scope of practice. According to the midwife specialists, the existing scope primarily guides the practice of registered midwives, rather than addressing the expanded roles of specialists. Historically, midwives practised under Regulation R.2598 of 1984, which outlines the scope of practice for nurses and midwives (SANC 1984). This regulation predates the introduction of the Training Regulation R.212 of 1993, which governs the education of midwife specialists (SANC 1993). The temporal gap between these two regulations may contribute to the misalignment between the training and practice frameworks. In 2022, Regulation R.2598 was revised and replaced by Regulation R.2127, which sets out the updated scope of practice for nurses and midwives (SANC 2022). However, the revised regulation does not explicitly include the roles and responsibilities of midwife and nurse specialists. As a result, while the current scope of practice provides legal protection for nurses and midwives, it may not adequately support or reflect the expanded functions of specialist practitioners.

The midwife specialists highlighted that regulations such as Regulation 2488, which are conditions under which midwives carry out their profession, are an extension of midwives’ scope of practice. The midwife specialists expressed that Regulation 2488 stipulates the conditions of practice for registered midwives (SANC 1990). Regulation 2488 was passed in 1990, 3 years before the training regulation R212 was promulgated (SANC 1990, 1993). These differences may justify the exclusion of midwife specialists from the conditions of midwives’ practice. The midwife specialists argued that they returned to their previous registered midwives’ roles. Unfortunately, this finding contradicts the SANC position paper that regards experienced and knowledgeable nurses and midwives as direct and indirect supervisors to the junior nursing and midwifery staff (SANC 2021a). This finding justifies the existing literature, which elucidates that the roles and responsibilities of midwife specialists need to be clearly defined in clinical facilities.

While the roles and responsibilities of the midwife specialists remain undefined by the SANC regulations 2127 and 2488, SANC recommended that the midwife specialists be allocated to the midwifery units to provide high-quality care. This finding may demonstrate the awareness of SANC regarding midwife specialists’ potential and support of midwife specialists as primary caregivers (Harper-Ovstebo & Mair 2024). The midwife specialists were, however, concerned that there are no specific regulations to differentiate their roles and responsibilities from the non-specialised midwives. Consequently, the midwife specialists experienced challenges assuming specialist roles, especially in the absence of physicians. This finding substantiates the existing literature striving to draw a distinction between the scope of practice of midwife specialists and registered midwives, which is challenging as there are no regulations from the Nursing and Midwifery Council (McKenna, Davis & Williams 2023).

While SANC supports the allocation of midwife specialists in midwifery units regarding position statements, it also cautions midwives against participating in invasive procedures as they contravene the scope of practice regulation 2127 (SANC 2022, 2021b). The midwife specialists expressed concern that they were trained in invasive midwifery interventions, which required teamwork between midwife specialists and physicians. This finding undermines the purpose of midwife specialist training, which was to enhance the knowledge and skills of midwife specialists (Altınayak, Apay & Vermeulen 2020). Additionally, this position statement restricts midwife specialists’ independent and interdependent professional roles.

The midwife specialists were aware of the legal restrictions of the current SANC practice regulations 2127 and 2488 during their encounters with complicated maternal and neonatal complications, which left the midwife specialists conflicted in applying their knowledge. Midwife specialists must practise in line with the legal and ethical framework. Ideally, midwife specialists are professionals who practise within the framework to avoid breaching the practice regulations through their acts. On the other hand, practising within the legal framework avoids intended and unintended omissions of the clinical interventions to the detriment of the patients, potentially resulting in litigation. This finding supports the existing literature on the increasing rate of litigation in maternity and neonatal units, accounting for close to 50% of the litigation bill (Miziara & Miziara 2022). Midwife specialists are held accountable for their acts and omissions and may be disciplined and sanctioned in Regulation 767, which prescribes disciplinary measures and sanctions (SANC 2014).

The midwife specialists recognise the need for more alignment between the education, training and practice regulations. Consequently, the midwife specialists recommended revising the practice regulations to avoid misalignment. A call for revising midwifery regulations to allow the autonomous and independent practice of midwife specialists is a global concern in the midwifery fraternity (Andraka-Christou et al. 2022). Other African countries, such as Ghana and Liberia, call for professional regulatory bodies to provide practice regulations to legalise advanced midwifery skills to prevent maternal and neonatal complications (Kpangaala-Flomo et al. 2021). The midwife specialists believed that revising the scope of practice would positively influence the job descriptions. The midwife specialists’ opinion is valid; the scope of practice serves as a framework of reference for developing the job descriptions to allow the practitioners to practise within the set parameters (Forsetlund et al. 2021). The midwife specialists echoed that revising the scope of practice is pertinent to differentiate the roles and responsibilities between midwife specialists and registered midwives. This finding may suggest that the midwife specialists seek professional identity and recognition from SANC, which is well explained in the literature (Kraienhemke 2021).

### Strengths and limitations

The qualitative study limited the sample size to 16 midwife specialists from the public health sector, excluding midwife specialists from the private sector. However, a similar study linked to the major doctoral study is underway in the private health sector.

Although the sample size was small, it comprised midwives from seven out of nine provinces, which suggests that the study’s findings indicate that the challenges the midwives face regarding the current legislative framework are limited to the public sector. Therefore, the study cannot be generalised to the South African context, but may be replicated.

### Recommendations

The study highlights the need for more alignment between education, training and practice legislative framework, which resulted in midwife specialists’ inability to utilise their knowledge and skills. Midwife specialists recommend revising and aligning the education, training and legislative framework. These findings also have the potential to alter the practice. Revising the scope of practice to be aligned with the midwife specialists’ curricula and competencies would enable midwife specialists to gain legal protection and the power to practise as specialists.

## Conclusion

The study highlights midwife specialist education and training as a legal and approved training programme in South Africa, as evidenced by the availability of training Regulation 635 and competencies for midwife specialists. These education and training regulations amplify the expanded knowledge, skills and clinical acumen as essential competencies for midwife specialists’ practice. The study’s findings acknowledge the presence of practice regulations 2127 and 2488 from SANC to guide the midwife specialist’s practice. However, the study elucidates the need for more alignment between education, training, practice and the legislative framework guiding the midwife specialists’ profession. Accordingly, these misalignments resulted in challenges in defining the midwife specialist as an independent and autonomous practitioner with roles and responsibilities. Consequently, the midwife specialists could not apply their clinical knowledge and skills to resolve patients’ complications.

Considering the challenges brought about by the misaligned legislative framework, the study recommends revising the scope of practice and conditions under which midwife specialists carry out their profession to include the knowledge and skills of midwife specialists. Revising the scope of practice is a point of departure in providing legal recognition to midwife specialists’ professional practice. Another study is underway to formulate strategies to serve as a framework for revising the scope of practice.
